# Non-Hodgkin's lymphoma of the sphenoid sinus presenting as isolated oculomotor nerve palsy

**DOI:** 10.1186/1477-7819-5-86

**Published:** 2007-08-03

**Authors:** Young Mok Park, Jun Hyung Cho, Jae Yong Cho, Ji Soon Huh, Jung Yong Ahn

**Affiliations:** 1 Department of Neurosurgery, Yonsei University College of Medicine, Seoul, Republic of Korea; 2 Department of Medical Oncology, Yonsei University College of Medicine, Seoul, Republic of Korea; 3 Department of Neurosurgery, College of Medicine, Cheju National University Hospital, Jeju, Republic of Korea

## Abstract

**Background:**

Solitary involvement of the sphenoid sinus has rarely been reported in non-Hodgkin's lymphoma. Isolated oculomotor nerve palsy is uncommon as an initial presentation of malignant tumors of the sphenoid sinus.

**Case presentation:**

A 53-year-old woman presented with a three-month history of headache and diplopia. Neurological examination revealed complete left oculomotor nerve palsy. Magnetic Resonance Imaging (MRI) demonstrated a homogenous soft-tissue lesion occupying the left sphenoid sinus and invading the left cavernous sinus. The patient underwent transsphenoidal biopsy and the lesion was histologically diagnosed as non-Hodgkin's lymphoma, diffuse large B-cell type. Tumor cells were positive for CD20 and negative for CD3. Following six cycles of chemotherapy, the left oculomotor nerve palsy that had been previously observed was completely resolved. There was no enhancing lesion noted on follow-up MRI.

**Conclusion:**

It is important to recognize that non-Hodgkin's lymphoma of the sphenoid sinus can present with isolated oculomotor nerve palsy, although it is extremely rare. The cranial nerve deficits can resolve dramatically after chemotherapy.

## Background

Approximately 10–34% of all non-Hodgkin's lymphomas arise from extranodal sites [1-3]. Of these, nasal or paranasal lymphomas account for less than 3% of all malignant extranodal lymphomas [4]. The sphenoid sinus is a rare primary site for extranodal lymphomas; only a few case reports exist in the literature [5-10]. Clinical signs and symptoms of malignant lymphomas of the paranasal sinuses include a mass in the nasal cavity, facial pain, paresthesia, recurrent sinusitis, nasal discharge, eyelid swelling, and proptosis if orbital invasion has occurred [10]. Isolated oculomotor nerve palsy is a rare complication of cavernous sinus invasion in non-Hodgkin's lymphomas.

We report a case of isolated oculomotor nerve involvement presenting in a woman with non-Hodgkin's lymphoma originating from the sphenoid sinus. This case demonstrates the resolution of oculomotor nerve palsy after chemotherapy.

## Case report

A 53-year-old woman presented to the Neurology outpatient office with a three-month history of headache and diplopia. There was no history of fever, weight loss, or nocturnal sweating. The patient had no history of diabetes, hypertension, or neurological diseases and no risk factors for stroke. No cervical bruits or lymphadenopathy in the cervical, supraclavicular or axillary areas were appreciated. Neurological examination revealed complete left occulomotor nerve palsy, with ptosis, mydriasis and outward positioning of the left eye. The remaining results of the physical examination were within normal limits. All serum laboratory and hormonal values were within the normal ranges.

**Figure 1:**
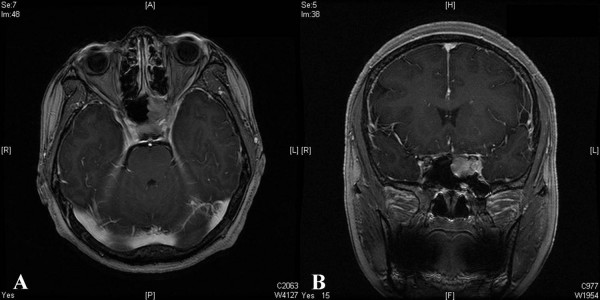
Axial (A) and sagittal (B) preoperative Gadolinium-enhanced MRI, demonstrating a homogenous soft-tissue lesion occupying the left sphenoid sinus and invading the left cavernous sinus.

MRI demonstrated a homogenous soft-tissue lesion occupying the left sphenoid sinus and invading the left cavernous sinus (Figure 1). The mass was homogenously enhanced by Gadolinium injection, and no intradural extension of the tumor was noted. A space-occupying lesion in the sphenoid sinus, such as a carcinoma, mucocele, or ectopic pituitary adenoma, was suspected as a preliminary diagnosis.

**Figure 2:**
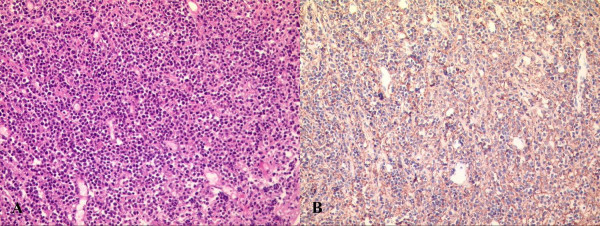
A: Photomicrograph demonstrating non-Hodgkin's lymphoma of the diffuse large B-cell type, which has uniform, round-to-oval nuclei with vesicular chromatin and one or multiple conspicuous nucleoli (H & E, original magnification × 200). B: These tumor cells are positive for CD20 (Original magnification × 200).

During the open surgery, the left sphenoid sinus contained a red friable vascular tumor, which was biopsied. Frozen sections of an intrasurgical biopsy were diagnosed as compatible with a small round cell tumor. The final histological diagnosis was non-Hodgkin's lymphoma, diffuse large B-cell type, which has uniform, round-to-oval nuclei with vesicular chromatin and one or multiple conspicuous nucleoli. These tumor cells were positive for CD20 and negative for CD3 (Figure 2).

**Figure 3:**
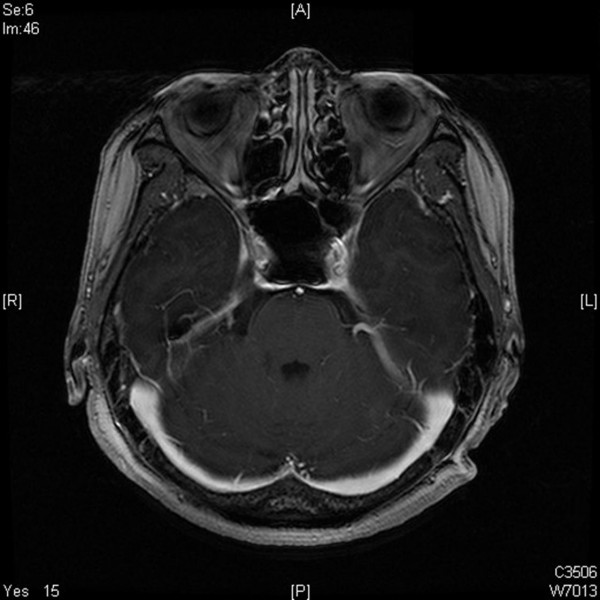
Following six cycles of chemotherapy, axial MRI revealed no enhancing lesion in the sphenoid and cavernous sinuses.

The patient was referred to medical oncology for a staging work-up, including bone marrow biopsy and positron emission tomography (PET), all of which were negative. The patient received chemotherapy consisting of eight cycles of CHOP (cyclophosphamide, adriamycin, vincristine (oncovin), and prednisone) with adjuvant Rituximab. Following six cycles of chemotherapy, the previously observed left third nerve palsy was completely resolved. There was no enhancing lesion noted on follow-up MRI 6 months postsurgery (Figure 3). The patient is currently under regular follow-up monthly in the medical oncology clinic.

## Discussion

This case study is unique in two aspects: the initial clinical presentation of isolated oculomotor nerve palsy without any additional neurologic deficits is rare, and the nature of the tumor, which occupied the sphenoid sinus, is a rarely documented site of non-Hodgkin's lymphoma. To our knowledge, there have been only six documented cases of primary sphenoidal non-Hodgkin's lymphoma in the literature. The clinical characteristics of these cases are summarized in Table 1. There have been a total of six males and one female documented in the literature, including our case. The median age was 48 years (range 5–78). Presenting symptoms included headache, visual disturbance and cranial nerve involvement.

**Table 1:** Clinical characteristics of patients with primary sphenoidal non-Hodgkin lymphoma previously described in the literature

Age (yrs)/sex	Clinical symptoms and signs	Local extension	Pathology	Treatment	Reference
52/M	Reduced visual acuity, diplopia, retroorbital pain Orbital apex syndrome with blindness	Left optic canal	Diffuse large B cell type	Radiotherapy Chemotherapy	Ueba et al [15]
39/M	Nasal congestion, facial pain, paresthesia	Sellar turcica	Diffuse large T cell type	Radiotherapy CHOP-M	Weber and Loewenheim [16]
44/M	Diplopia, bilateral abducens nerve palsy	Clivus, cavernous sinus, ethmoid sinus	Diffuse large B cell type	CHOP-M	Deleu et al [3]
78/M	Diplopia, abducens nerve palsy	Clivus, cavernous sinus	Diffuse large B cell type	Radiotherapy (45 Gy)	Ferrario et al [4]
5/M	Sudden visual loss, optic neuropathy	Suprasellar cavernous sinus	Diffuse large B cell type	Radiotherapy (60 Gy) Chemotherapy*	Roth and Siatkowski [12]
64/M	Headache, diplopia, oculomotor nerve palsy	Sellar turcica, cavernous sinus	Diffuse large B cell type	COPPA-M Radiotherapy (50 Gy)	Metellus et al [8]
53/F	Headache, ptosis, oculomotor nerve palsy	Cavernous sinus	Diffuse large B cell type	CHOP-R	Present case

* CHOP-M = cyclophosphamide, adriamycin, vincristine, prednisone, and methotrexate; CHOP-R = cyclophosphamide, adriamycin, vincristine, prednisone, and rituximab; COPPA-M = cyclophosphamide, Oncovin, procarbazine, prednisone, adriamycin, and methotrexate.

All reported cases had cranial nerve deficits with the sixth cranial nerve being the nerve most commonly affected [5,6]. In fact, diplopia secondary to sixth nerve palsy is one of the earliest signs of a diseased sphenoid. This involvement is attributed to the long, medial intracavernous position of the abducens. Lawson *et al*., [11] reported sixth nerve involvement in 6% of inflammatory cases and in 50% of neoplastic cases of isolated sphenoid sinus disease, with equal incidence for both benign and malignant tumors. The oculomotor nerve was the second most affected, as demonstrated by our case [7]. Involvement of the trigeminal nerve is via the first and second divisions, producing retrobulbar pain and midfacial pain and numbness [10]. With regard to the total incidence of objective cranial nerve deficits, these symptoms are directly related to the extent of the lesions.

Radiological imaging is vital in many aspects, including assessment of tumor extension, assessment of bony destruction, evidence of mucosal thickening, and choice of the best biopsy site and route. Although computed tomography (CT) is the best technique to demonstrate fine bony detail, MRI can adequately assess most areas of bony destruction and has a number of additional advantages. MRI can also more easily distinguish a tumor from mucosal thickening or retained sinus secretions [12]. Extension into surrounding spaces and into the cranial fossa is more easily evaluated in coronal and sagittal images. Bone remodeling and mucosal thickening are associated with chronic inflammatory disease, especially if a mucocele is present. Bone erosion is found principally with neoplastic disease, occurring rarely with mucoceles [11]. Extension is associated with malignant tumors. The reported isolated sphenoid sinus disease incidence is listed in Table 2[11,13-15]. Non-Hodgkin's lymphomas involving the sphenoid sinus are rarely considered in the differential diagnosis of a mass involving the sphenoid sinus without other systemic manifestations such as weight loss, night sweating or fever. This is probably due to the exceedingly rare incidence of this disease.

**Table 2:** Classification of isolated sphenoid sinus disease

Inflammatory disease	Benign neoplasms	Malignant neoplasms
Sinusitis acute/chronic	Inverting papilloma	Squamous cell carcinoma
Mucocele	Pseudotumor	Adenoid cystic carcinoma
Polyps retention cyst	Myxofibroma	Chondrosarcoma
Fungal sinusitis	Schwannoma	Neuroendocrine carcinoma
	Osteochondroma	Mucoepidermoid carcinoma
	Plasmacytoma	Malignant fibrous histiocytoma
	Chordoma	Osteogenic sarcoma
	Pituitary adenoma	Lymphoma
	Meningioma	Olfactory neuroblastoma
	Epidermoid tumor	Ewing's sarcoma
	Cavernous hemangioma	Giant cell tumor
		Metastatic tumors

It is unlikely for T-lymphomas to involve the nasal cavity because most paranasal lymphomas are B-cell tumors [16,17]. Only one report exists on the histological and clinical features of sphenoid sinus T-cell lymphoma [10]. Others studies, including our case, were confirmed as diffuse, large B-cell lymphomas. The primary site of the lymphoma together with its histologic grade, T/B phenotype and clinical stage may be important prognostic factors in primary non-Hodgkin's lymphoma of the sinonasal cavities [18]. These lymphomas have a poor prognosis which is usually worse than that associated with lymphomas in other sites in the body [19].

Non-Hodgkin's lymphomas are frequently treated with, and respond to, a combination of chemotherapy and radiotherapy. A review of several reports suggests that the best treatment outcomes are obtained with the CHOP (cyclophosphamide, adriamycin, vincristine (oncovin), and prednisone) regimen, given at three-week intervals [20]. Rituximab is a therapeutic antibody directed against the CD20 surface antigen, which is frequently present in lymphoma cells. Its use in conjunction with CHOP augments a treatment response in lymphomas expressing the CD20 antigen, as seen in our case [21]. Chemotherapy is frequently followed by loco-regional radiotherapy at a dose of 30 to 40 Gy [15]. In our case the oculomotor nerve palsy was dramatically improved over the course of chemotherapy.

## Conclusion

To our knowledge, there have been only six documented cases of primary sphenoidal non-Hodgkin's lymphoma. It is important to recognize that non-Hodgkin's lymphoma of the sphenoid sinus can present with isolated oculomotor nerve palsy, although it is extremely rare. The cranial nerve deficits can be dramatically resolved following chemotherapy.

## Competing interests

The author(s) declare that they have no competing interests.

## Authors' contributions

YMPconceptualized the study, gathered the data, and drafted the manuscript. JHC performed the literature search and helped to draft the manuscript. JYC participated in chemotherapy and took charge of post-operative management together with JYA. JYA supervised the process and finally approved the manuscript for publication. JSH involved in manuscript revision.

All authors have read and approved the final manuscript.
